# Postoperative Fasting Blood Glucose Predicts Prognosis in Stage I-III Colorectal Cancer Patients Undergoing Resection

**DOI:** 10.1155/2020/2482409

**Published:** 2020-01-08

**Authors:** Rui Xu, Junhao You, Fang Li, Bing Yan

**Affiliations:** Department of Oncology, Hainan Hospital of PLA General Hospital, Sanya city, Hainan province 572000, China

## Abstract

**Purpose:**

The relationship between high blood glucose and colorectal cancer (CRC) has been studied, but the role of postoperative fasting blood glucose (FBG) in patients with a prior normal FBG has never been addressed.

**Methods:**

A total of 120 CRC patients staged I-III were enrolled, and the prognostic value of postoperative FBG for disease-free survival (DFS) was determined by Kaplan-Meier analysis. Univariate and multivariate analyses were conducted to test other clinicopathological parameters, including preoperative hemoglobin (HGB) and the neutrophil-lymphocyte ratio (NLR).

**Results:**

By a cut-off point of 5.11 mmol/L, 51 and 69 patients were divided into low postoperative FBG (<5.11 mmol/L) and high postoperative FBG (≥5.11 mmol/L) groups, respectively. A high postoperative FBG was more common in older age (*P* = 0.01), left-located tumor (*P* = 0.02), smaller tumor diameter (*P* = 0.01), node negative involvement (*P* = 0.01), lesser positive lymph nodes (*P* = 0.02), and high preoperative HGB (*P* = 0.01). Further, high postoperative FBG patients displayed a significantly better DFS than low postoperative FBG patients (48.80 ± 22.12 months *vs*. 40.06 ± 24.36 months, *P* = 0.04), but it was less likely to be an independent prognostic factor.

**Conclusions:**

Postoperative FBG plays a temporal prognostic role for patients with stage I-III CRC with a prior normal FBG, but it is not an independent prognostic factor.

## 1. Introduction

The relationship between blood glucose metabolism and cancer has been under extensive study for many years, particularly for those diagnosed with type 2 diabetes mellitus (T2DM) [1]. In some epidemiological studies, the high morbidity of malignancies in individuals with long-term aberrant fasting blood glucose (FBG) or T2DM has been established [2, 3], and examples have been provided in breast cancer [3], esophageal cancer [4], liver cancer [5], and colorectal cancer (CRC) [3, 6], which is one of the leading causes of cancer-related death in China [7].

Notably, in addition to high FBG promoting cancer aggression [8], it is also associated with a poor prognosis for patients [9, 10]. For example, Contiero et al. reported a study that included 1,261 stage I-III breast cancer patients and found that high FBG correlated with not only distant metastasis or recurrence but also death [9]. Luo et al. also performed a study with 342 non-small-cell lung cancer (NSCLC) patients and found that high FBG was linked to a 69% excess risk of all-cause mortality [10]. However, in contrast, Cui et al. conducted a study of 391 patients with CRC, including 116 patients with high FBG, and found that FBG was linked to larger tumor diameters, lower tumor differentiation, advanced TNM stage, and a more ulcerative type but had no influence on distant metastasis or overall survival (OS) [11]. Interestingly, cancer treatment approaches can also affect FBG, and typical examples have been seen in breast or prostate cancer sufferers who receive endocrine therapies [12, 13]. In addition, it has been reported that surgery also improves glucose metabolism in pancreatic cancer patients with prior T2DM [14]. Nonetheless, studies concerning the role of FBG in CRC patients treated with curable resection, particularly in those with a prior normal FBG, have not been reported.

Although it is mainly regulated by insulin, the level of FBG in individuals has been found to be closely related to some characteristics, including body mass index (BMI) [15, 16] and the neutrophil-lymphocyte ratio (NLR). The NLR was found to be positively associated with FBG in patients with T2DM or high FBG [17] but was negatively correlated with FBG in normal subjects [18]. In this study, we aimed to explore the prognostic role of postoperative FBG and other clinicopathological features, including the abovementioned BMI and NLR, in stage I-III CRC patients with a prior normal FBG.

## 2. Materials and Methods

### 2.1. Patient Enrollment

From January 2011 to October 2014, 120 patients with colorectal adenocarcinoma (according to the 7^th^ edition of the American Joint Committee on Cancer Staging) staged I-III were collected at Hainan Hospital of PLA General Hospital. Patients with the following criteria were excluded: (1) age < 18 years old; (2) a history of previous T2DM or elevated FBG beyond the upper limit (ref: 3.4-6.1 mmol/L); (3) multiple or recurrent malignancies or *in situ* lesions; (4) a history of previous neoadjuvant therapy; (5) complications such as infection, obstruction, and bleeding after surgery; (6) unsuccessful oral feeding within 14 days after the operation; and (7) no record of postoperative FBG or a follow-up date. Clinicopathological parameters included age (<60 or ≥60 years old), sex, BMI, CEA (ref: 0-5 *μ*g/L), and CA19-9 (ref: 0.1-37 *μ*g/mL) values, and the results of routine blood tests including hemoglobin (HGB; males: 137-179 g/L and females: 116-155 g/L), absolute white blood cell (WBC) counts (ref: 3.5-10^9^/L), monocyte (MON) counts (ref: 0.10-0.8^9^/L), platelets (PLT; 100-300^9^/L), and serum albumin (ALB; ref: 35-50 g/L) were recorded before the operation. In addition, pathological results regarding tumor location, histological grade, invasive depth, maximum tumor diameter, etc., were recorded. Postoperative adjuvant therapies were recorded. The study was supervised by the ethics committee of Hainan Hospital of PLA General Hospital (approved ID: 301HLFYLL15), and written informed consent was not needed since this was a retrospective study.

### 2.2. Determination of Preoperative/Postoperative FBG and NLR

Routine laboratory tests were performed between 6:00 and 9:00 am on peripheral venous blood within 1 month before surgery (preoperative) and at least 14 days to 1 month after surgery (postoperative). In addition, FBG values in patients with available records 3-6 months after the operation were also collected. The BMI and NLR were determined as previously described [19, 20].

### 2.3. Follow-Up Procedure and Definition of Disease-Free Survival (DFS)

Patient follow-up was achieved by telephone or a visit to the medical records department at the hospital at intervals of 3-6 months for the first 3 years and 6-12 months for the next year. DFS was defined as the point from the date of operation until the date of first recurrence or death from any cause. The primary study endpoint was 3 years DFS, as defined in a previous study [21], and the last follow-up point occurred in October 2019.

### 2.4. Statistical Analysis

All the statistical analyses were conducted by using SPSS 20.0 software (SPSS Inc., Chicago, IL, USA). Receiver operating characteristic (ROC) curve analysis was used to determine the optimal cut-off value for FBG, and its relationship with other clinicopathological parameters was calculated by the *χ*^2^ test, Fisher's exact test, Student's *t*-test, or the Mann–Whitney *U* test when appropriate. Kaplan-Meier (K-M) survival curves were constructed to compare patients with low and high FBG, and significant differences were determined by the log-rank test [9]. Univariate and multivariate analyses were conducted by using the Cox proportional hazards model [22]; the proportional hazards assumption was checked by Schoenfeld residuals for continuous variables [9] or by K-M for categorical variables. A double-sided *P* < 0.05 was considered statistically significant.

## 3. Results

### 3.1. Demographic Characteristics and Differences in Postoperative FBG according to Various Clinicopathological Parameters

**Figure 1:**
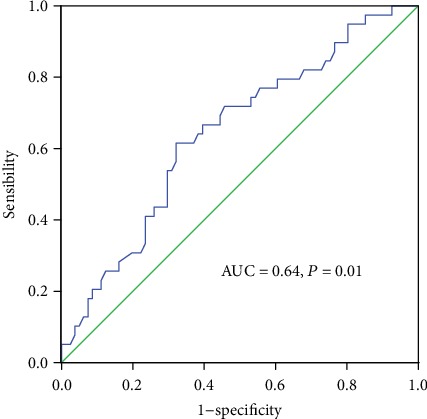
Receiver operating characteristic curve analysis of postoperative FBG in patients.

In total, 40 female and 80 male patients were included, and the mean age of the patients was 57.87 years old (range: 24-85 years old), with a medium follow-up time of 45.08 months (range: 1-81 months). There were 21, 53, and 46 patients with stage I, II, and III disease, respectively. As shown in Figure 1, with a cut-off point of 5.11 mmol/L, postoperative FBG had a sensitivity and specificity of 38.50% and 32.10%, respectively, in predicting DFS (AUC = 0.64, *P* = 0.01). When patients were divided into low or high groups according to this cut-off point, a relatively high postoperative FBG could be found in older age (*P* = 0.01), left-located tumor (*P* = 0.02), smaller tumor diameter (*P* = 0.01), node negative involvement (*P* = 0.01), lesser positive lymph nodes (*P* = 0.02), and high preoperative HGB (*P* = 0.01) (Table 1). In addition, 80 patients were available for the postoperative 3-6 months FBG, and 47 maintained a high FBG compared to their preoperative values. In these selected cases, the postoperative FBG failed to display a significant role in predicting DFS (AUC = 0.40, *P* = 0.15). (Data not included in Table 1).

### 3.2. Predictive Value of Postoperative FBG for DFS

**Figure 2:**
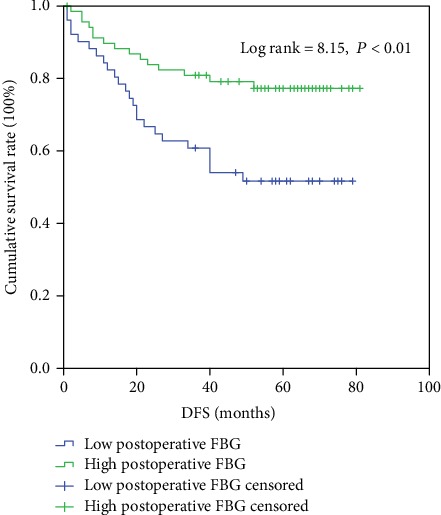
Impact of low or high postoperative FBG on disease-free survival.

According to K-M analyses, we then examined the predictive value of postoperative FBG for DFS. As shown in Figure 2, patients with a high postoperative FBG displayed a significantly better DFS than those with a low postoperative FBG (48.80 ± 22.12 months *vs*. 40.06 ± 24.36 months, *P* = 0.04).

### 3.3. Univariate and Multivariate Analyses for the Factors Correlated with DFS

As shown in Table 2, according to univariate tests, preoperative CEA and CA19-9 levels, invasive depth, maximum tumor diameter, node involvement, number of positive nodes, stage III, BMI, preoperative NLR, ALB, and postoperative FBG correlated with the DFS. According to the HRs obtained, it can be seen that the majority of the above factors played as a risk factors for DFS except ALB and postoperative FBG. A *P* < 0.05 was used as a cut-off value in multivariate analysis after checking the proportional hazards assumption; the preoperative CEA level, invasive depth, node involvement, and preoperative NLR and ALB were found to be independent prognostic factors; according to the HRs obtained, the invasive depth was the most significant risk factor and only the ALB was a protective factor for the DFS.

## 4. Discussion

In this study, we found that high postoperative FBG predicted a better DFS for CRC than a low postoperative FBG. In addition, high postoperative FBG correlated with parameters including age, tumor location, maximum tumor diameter, number of positive nodes, and preoperative HGB and NLR as well as a prolonged DFS. Although according to the Cox proportional hazards model, the level of postoperative FBG was less likely to be an independent prognostic factor, these data provide, to the best of our knowledge, the first observation concerning the prognostic role of postoperative FBG in CRC, in particular, within patients with a prior normal FBG.

Postoperative FBG has been reported to play a significant prognostic role in cancer patients. For example, Yang et al. carried out a prospective cohort study with 387 stage I-IV NSCLC patients and found that patients with a low FBG (<4 mmol/L) had a significantly higher risk of death than those with a high FBG [23]. Wu et al. conducted a study that included 306 stage 0-III esophageal cancer patients who underwent esophagectomy and found that low postoperative FBG was related to poor survival, and an FBG ≤4 mmol/L was independently linked to poor survival [16]. In our study, we selected patients without a background of a prior preoperative aberrant FBG and found that a low postoperative FBG (<5.11 mmol/L) was associated with poor survival for CRC, which was partially consistent with the results of these studies [16, 23]. Notably, we also found that 58.75% (47/80) of patients maintained a relatively high FBG compared to their preoperative value at the 3-6 m follow-up, but FBG failed to present any prognostic value for DFS. Although the study sample size was relatively small, the importance of longitudinal tests of FBG to predict the DFS in patients is important.

Until now, the underlying mechanisms of glucose metabolism in cancer patients were still not fully understood, but glucose metabolism was potentially correlated with some characteristics. In our study, postoperative low FBG was correlated with younger age, disease on the right side of their body, large tumor diameter, more positive nodes, and low preoperative HGB. It is notable that some of these parameters are well-established prognostic factors for CRC. For example, studies have indicated that right-sided tumors have an inferior prognosis in terms of OS in stage III tumors [24] and in those undergoing curative resection of liver metastases [25]. Additionally, a large tumor size was found to be associated with poor OS in those receiving chemotherapy [26]. Additionally, other studies have indicated that younger age [27] and low HGB are associated with poor prognosis in patients [28]. We speculate that these parameters contribute to the link between poor DFS and high postoperative FBG in CRC.

Although studies have indicated that high glucose levels can not only accelerate tumorigenesis [29] but also promote cancer aggression [8], there are still conflicting results. For example, two studies indicated that hyperglycemia inhibited malignant cell spread and metastasis in patients with cancer such as NSCLC [30, 31]. In line with these findings, some experimental studies have indicated the consequences of glucose deprivation in CRC. For example, Li et al. indicated that in the human colon cancer cell line HT-29, glucose deprivation increased cell proliferation by 30% when cells were exposed to *γ*-radiation [32]. Hu et al. reported that glucose deprivation resulted in chemoresistance in CRC cells by upregulating transcription factor 4 expression [33]. Additionally, Nishimoto et al. found that glucose deprivation played a central role in the acquisition of antiapoptotic mechanisms by human colorectal cancer cells via activation of hypoxia-inducible factor-1*α* [34]. Recently, a study indicated that cancer dissemination occurred even when the primary lesions were clinically undetectable [35]. However, whether high glucose levels could inhibit remaining cancer cells in patients undergoing curative surgery based on the findings of the above studies is still largely unknown, and more clinical studies are needed in the future.

The present study had many limitations. First, its small sample size may limit the statistical power. Second, taking into consideration the complexity of glucose metabolism in cancer patients, some potential residual confounders, for example, patients received adjuvant therapies would have different glucose metabolism than their counterparts, that may bias the findings. Third, a more prolonged follow-up duration would have allowed the role of postoperative FBG in predicting OS to be determined. Nonetheless, more convincing evidences can only be obtained from prospective randomized controlled studies and fundamental researches in this field in the future.

## 5. Conclusion

Overall, our results indicate that postoperative FBG plays a temporal prognostic role for stage I-III CRC patients with a prior normal FBG, but FBG was likely not an independent prognostic factor.

## Figures and Tables

**Table 1:** Differences in postoperative FBG among different clinicopathological parameters.

	No. of patients	Postoperative FBG		
		Low	High	*P*
Age (y)				**0.01**
<60	62	33	29	
≥60	58	18	40	
Sex				0.43
Female	40	15	25	
Male	80	36	44	
Tumor location				**0.02**
Right	34	20	14	
Left	86	31	55	
Histological grade				0.07
Well	4	0	4	
Moderate	90	36	54	
Poor	26	15	11	
CEA level				0.13
Normal	82	31	51	
Elevated	38	20	18	
CA19-9 level				0.08
Normal	102	40	62	
Elevated	18	11	7	
Invasive depth				0.36
T_1+2_	26	9	17	
T_3+4_	94	42	52	
Maximum tumor diameter (cm)		5.04 ± 2.18	4.14 ± 1.79	**0.01**
Node involvement				**0.01**
N_0_	72	24	48	
N_1+2_	48	27	21	
Positive nodes	120	2.94 ± 5.55	1.46 ± 3.24	**0.02**
TNM stage				0.05
I	21	5	16	
II	53	21	32	
III	46	25	21	
Adjuvant therapies				**0.04**
Received	70	35	35	
None	50	16	34	
BMI (kg/m^2^)	120	22.87 ± 4.18	23.66 ± 3.27	0.33
Preoperative measures				
HGB (g/L)	120	119.27 ± 18.95	127.24 ± 12.55	**0.01**
WBC (×10^9^/L)	120	6.29 ± 1.84	6.45 ± 1.77	0.64
NLR	120	2.66 ± 1.93	2.06 ± 1.20	0.11
MON (×10^9^/L)	120	0.49 ± 0.20	0.50 ± 0.17	0.87
PLT (×10^9^/L)	120	245.35 ± 86.91	250.22 ± 98.50	0.96
ALB (g/L)	120	39.42 ± 5.04	39.86 ± 2.90	0.85

**Table 2:** Univariate and multivariate analyses of different parameters for prognosis by Cox proportional hazards model.

	No. of patients	No. of events	Univariate			Multivariate		
			*P*	HR	95% CI	*P*	HR	95% CI
Age (years)								
<60	62	19	1					
≥60	58	20	0.73	1.12	0.60-2.09			
Sex								
Female	80	13	1					
Male	40	26	0.96	1.02	0.52-1.98			
Tumor location								
Right	86	8						
Left	34	31	0.27	1.55	0.71-3.37			
Histological grade								
Well	26	10	1					
Moderate+poor	94	29	0.32	0.70	0.34-1.43			
CEA level								
Normal	82	17	1			1		
Elevated	38	22	**<0.01**	3.85	2.04-7.28	**<0.01**	2.85	1.45-5.61
CA19-9 level								
Normal	102	28	1					
Elevated	18	11	**<0.01**	3.14	1.56-6.33			
Invasive depth								
T_1+2_	26	2	1			1		
T_3+4_	94	37	**0.01**	5.93	1.43-24.61	**0.03**	5.04	1.18-21.45
Maximum tumor diameter (cm)	120	39	**<0.01**	1.24	1.07-1.43			
Node involvement								
N_0_	72	16	1					
N_1+2_	48	23	**<0.01**	2.64	1.39-5.01			
Positive nodes	120		**<0.01**	1.17	1.11-1.24	**<0.01**	1.11	1.04-1.18
TNM stage			**<0.01**	2.39	1.43-4.01			
I	21	1	1					
II^a^	53	16	**0.06**	6.91	0.92-52.12			
III^b^	46	22	**0.01**	12.86	1.73-95.52			
Adjuvant therapies								
Received	70	27	1					
None	50	12	0.10	0.57	0.29-1.12			
BMI (kg/m^2^)	120	39	**0.04**	0.91	0.83-0.99			
Preoperative measures								
WBC (×10^9^/L)	120	39	0.68	1.04	0.87-1.23			
HGB (g/L)	120	39	0.71	1.00	0.98-1.02			
NLR	120	39	**<0.01**	1.44	1.24-1.67	**<0.01**	1.35	1.16-1.58
PLT (×10^9^/L)	120	39	0.61	1.00	1.00-1.00			
ALB (g/L)	120	39	**<0.01**	0.88	0.81-0.96	**<0.01**	0.90	0.83-0.97
Postoperative FBG (mmol/L)	120	39	**0.01**	0.56	0.34-0.88			

^a, b^Compared with stage I.

## Data Availability

The data used to support the findings of this study are available from the corresponding author upon request.
